# Editorial

**Published:** 1985-04

**Authors:** 


### Editorial

### SALUTE TO THE BORN-AGAIN BLACK BAG

The Bristol Medico-Chirurgical Journal rejoices that its little sister 'The Black Bag' has reappeared. She was born in 1937 in the shadow of Hitler's War that was to come. Significantly the first article was entitled 'The first gas attack in April 1915' and was an account by an M.O. faced with the casualties on that horrific occasion. However, all was not gloom. The Editor pointed out to his fellow students that they now had an opportunity to respond to some of the humour meted out 'at our expense' by staff who 'in lecture rooms and wards hold the monopoly', and he added 'For those who may be victimised in these pages we trust you will find it a pleasant tonic and that it will never leave behind a nasty taste'. Not surprisingly perhaps, the name of the Editor or of his colleagues in the enterprise is not revealed in any number until 1939, by which time any victimisations that had appeared could certainly have been no more than a 'pleasant tonic'. He was then named as M. O. Skelton and to him must be given the major credit. Dr. Martin Skelton, now retired, has since had a distinguished career as a consultant pathologist and through these pages sends greetings and good wishes to his successors. The original number contains two excellent caricatures, one of a gentleman from behind with protruding ears and wearing morning tails over the title 'I well rememburr'. He is identified as Professor Rendell Short and Dr. Skelton confirms that morning tails were his regular apparel even when riding his bicycle. The other is of a rather wild looking young man in profile with a prominent lower lip and wearing a stethoscope over the title 'Perry carditis' who surely needs no further identification. The Black Bag described itself as the Journal of the Medical Faculty of Bristol University, but also stated that it owed its existence to the Galenicals, the Student Society which had been started in 1934. It was not in fact the first Bristol medical student journal, in an Ave note, Professor Rendell Short wrote 'I well remember its predecessor in my own student days, called the Stethoscope, which had a very successful run for many years'. By April 1940 four excellent numbers had been produced, with student contribution tending to be anecdotal and poetic, interspersed with weightier material from staff.

There was a wartime gap between 1940 and 1945 when a 'Utility wartime model' appeared in typescript. The original Black Bag had staff as well as students on the Editorial committee, this time it was to be a 'student magazine, written by students for students, and the contents will vary from the ridiculous to the sublime'. In June 1946 a Black bag appeared essentially similar in design to the 1937 original. It continued until 1972, producing a number each term, and then abruptly ceased. Cause of death? – still uncertain. There had been no previous symptoms to suggest underlying illness, such as emaciation or loss of vitality, no obvious cause of malnutrition such as loss of advertisements. Probably one has to ascribe the demise to cardiac arrest due to lack of a volunteer editor with a heart for the task.

Leafing through these pages, now bound into volumes in the Medical School Library is of absorbing interest. A lot of important history is contained in them, much solid material of an improving nature is there as well as humour, high and low. There are some extraordinarily good cartoons, some of the humorous verse is quite brilliant, one such gem printed in 1947 your editor was delighted to find appeared over the initials M.B.L. – realising that they belonged to Dr. Michael Lennard, now a member of our Editorial Committee. The effort required to keep a journal going is considerable, particularly if it is over and above the daily round, and especially so for a student who has to keep his sights on the next examination. It is very good to see a reborn Black Bag and a promising first number. The new Editorial Committee has adopted the very sensible policy of nominating an Editor for one number only, with a successor for the next number. In this way no one is inhibited from taking on a task that could become an incubus, and the succession is assured if new members are coopted to replace those who leave on graduation. The first Editor and prime mover in the new era is Christopher Spencer Jones, now in India. The Editor of the next number, now doing Pathology, is Mary Clare Bromage. We salute their initiative and their enterprise and we wish them luck.

### OUR IMAGE

"Aren't you playing God doctor?" must be a question that is hard to bear, especially when asked in front of the Television cameras of a clinician who has just had to make an agonising decision – as for example in the recent Oxford case, to discontinue dialysis of a patient who was deteriorating in spite of it, and whose disruptive behaviour was affecting the care of other patients.

Professor Stirrat's contribution 'From our Correspondents' (p. 43) discusses the increasingly hostile attitude taken to us. This is typified by the media men, who seem so often determined to provoke. One sometimes wonders if B.B.C. really stands for Broadcasting Begets Confrontation. Paradoxically the taunt of aspiring to divinity is not thrown at us by the Clerics, and when the media inquisitors use it, it is precisely when we cease to play God that we are accused—at the moment when we say 'the struggle is hopeless – Thy will be done'.

LEGAL AID

The Editor would like to acknowledge that the scholar who produced the elegant translation of the epigram of Lucilius (180–102 BC) that was the motto of this Journal for the first 69 years of its existence, and has now been readopted, is the Bristol Solicitor Mr. John Griffiths. He translated this motto 'Scire est nescire, nisi id me scire alius sciret' as 'What I know remains unknown, unless by me to others shown'. Since then Mr Griffiths has contributed a biographical article on that remarkable man Dr. Thomas Dimsdale, now almost forgotten, who before Jenner (1767) practised immunisation against small pox by variolation on a large scale.

Mr. Griffiths has put us further in his debt by discussing the implications for the medical practitioner of the decision of the Court of Appeal in December 1984 in 'Gillick v. West Norfolk and Wisbech Area Health Authority and another'. His article appears below and deals with the legal situation which results from this decision.

BRAVE NEW WORLD

The doctor faced with a girl under 16 asking for contraceptives and refusing to allow her parents to be consulted has to navigate a moral and emotional minefield as well as a legal one, and needs all the help he can get and we are grateful to Mr. Griffiths for his article. The extent of this minefield may not be realised by many of the older generation, but mores have changed to an extent that is hard for us to grasp. For typical case histories and comment see Sunday Telegraph, February 24th, 'When sex life begins at 14' by Graham Turner.

We seem to have escaped Orwell's dire predictions for 1984 but gradually the Brave New World of Aldous Huxley is creeping up on us.

We should like to congratulate our President Elect, Dr. Joze Jancar on his nomination to receive the Distinguished Achievement Award of the International Association for the Scientific Study of Mental Deficiency in the area of scientific literature for 1985. The presentation was made to him at the 7th World Congress of the Association in New Delhi in March.

